# A brief history of the development of transcranial tissue Doppler ultrasound

**DOI:** 10.1098/rsfs.2024.0031

**Published:** 2024-12-06

**Authors:** Jennifer K. Nicholls, Andrea Lecchini-Visintini, Jonathan Ince, Edward Pallett, Jatinder S. Minhas, Mitsuhiro Oura, Emma M. L. Chung

**Affiliations:** ^1^ Department of Cardiovascular Sciences, Cerebral Haemodynamics in Ageing and Stroke Medicine (CHiASM) Research Group University of Leicester, Leicester LE1 5WW, UK; ^2^ University Hospitals of Leicester NHS Trust, Leicester LE1 5WW, UK; ^3^ School of Electronics and Computer Science, University of Southampton, Southampton SO17 1BJ, UK; ^4^ National Institute for Health Research Leicester Biomedical Research Centre, University of Leicester, Leicester LE5 4PW, UK; ^5^ Nihon Kohden Corporation Tokorozawa-shi, Saitama 359-0037, Japan; ^6^ Department of Women and Children’s Health, School of Life Course and Population Sciences, Faculty of Life Sciences and Medicine, King’s College London, London SE1 7EH, UK

**Keywords:** brain tissue pulsations, brain tissue pulsation amplitude, transcranial doppler, transcranial tissue doppler, ultrasound

## Abstract

This article documents the early development of the first transcranial Doppler (TCD)-based ultrasound system for continuous monitoring of brain tissue pulsations (BTPs). Transcranial tissue Doppler (TCTD) uses a lightweight, wearable single-element ultrasound probe to track tissue motion perpendicular to the skin’s surface, providing tissue displacement estimates along a single beam line. Feasibility tests using an adapted TCD system confirmed that brain tissue motion data can be obtained from existing TCD hardware. Brain Tissue Velocimetry (Brain TV), a TCTD data acquisition system, was then developed to provide a lightweight and portable means of continuously recording TCTD data in real-time. Brain TV measurements are synchronized to a 3-lead electrocardiogram and can be recorded alongside other physiological measurements, such as blood pressure, heart rate and end-tidal carbon dioxide. We have shown that Brain TV is able to record BTPs from sample depths ranging from 22 to 80 mm below the probe’s surface and from multiple positions on the head. Studies in healthy volunteers, stroke patients and ultrasound phantom brain models demonstrate how TCTD might provide insights into the relationships between physiological measurements and brain tissue motion and show promise for rapid clinical assessment and continuous monitoring of BTPs.

## 1. Introduction

Transcranial Doppler (TCD) ultrasound is an established blood flow measurement technique for investigating cerebrovascular haemodynamics in stroke patients, cerebral autoregulation assessment and embolus detection. TCD-based brain tissue motion measurement devices have the potential to offer significant benefits over image-based motion measurement techniques in terms of cost, size, portability, patient comfort and suitability for physiological monitoring. Transcranial tissue Doppler (TCTD) measurements of brain tissue motion can be readily obtained through minor modifications to existing TCD hardware. As the relationship between traditional physiological measurements (such as blood flow, blood pressure (BP), heart rate (HR) and expired carbon dioxide [CO_2_]) has already been extensively studied, extending this knowledge to encompass interpretation of TCTD findings may provide an accelerated route to understanding the physiological factors affecting brain tissue motion.

This article aims to introduce and document initial experiences and key findings during the development of a TCD-based approach to measuring brain tissue motion. We provide an overview of early feasibility data obtained using a modified Spencer Technologies TCD system, as well as detailing the development of an initial TCTD research prototype (Brain Tissue Velocimetry [Brain TV]) designed to facilitate physiological measurement studies to understand the impact of changes in cerebrovascular physiology on brain tissue pulsations (BTPs). Findings in healthy volunteers, patients with acute ischaemic stroke (AIS), and in an ultrasound phantom brain model are used to illustrate the potential of TCTD for physiological measurement studies and clinical research.

## 2. Summary of the BTP literature

The brain is a soft tissue mass, surrounded by cerebrospinal fluid (CSF), and contained within a fixed-volume skull. The total combined volume of the brain, CSF and intracranial blood is expected to remain constant over the cardiac cycle; this hypothesis is known as the Monro−Kellie doctrine [1]. According to the Monro−Kellie doctrine, an increase in volume of one compartment in the brain (e.g. arterial blood volume during systole) should be mirrored by an equivalent decrease in volume of the other two compartments ([1]; via CSF displacement or a decrease in brain tissue volume). Over the cardiac cycle, blood initially enters the circulation during systole at a faster rate than it leaves through the veins [2], causing pressure within the major arteries to increase. Arteries are thought to expand between by a fraction of a percent depending on location and elasticity [3]. Once surplus blood transfers into the venous vasculature during diastole, the vessels return to their original size.

However, cardiac pulsations are not the sole contributor to brain tissue motion and motion within the brain is far from uniform; tissue properties [4], intracranial pressure (ICP) [5], respiration [2,6] and vasomotion [2] are also thought to play a role in explaining variations in brain tissue pulsatility (BTP); these impact brain motion regionally and on various timescales [2]. Brain tissue and CSF are displaced due to varying intrathoracic pressure generated during respiration, which also results in changes to venous blood volume [6]. Both cardiac and respiratory pulsations are thought to drive the clearance of waste solutes from the brain in CSF flow [7], contributing to CSF-driven pulsations [8]. Spontaneous vasomotion, which causes slow fluctuations in arterial smooth muscle tone [9], alters blood flow and is thought to occur somewhat independently from cardiac pulsatility.

Weaver *et al.* [10] previously used phase-contrast magnetic resonance imaging (MRI) to characterize and quantify motion of the brain, dividing the brain into four regions and calculating average displacement [10]. The largest brain motion was found close to the circle of Willis (approx. 150 µm) and weaker brain pulsations were identified at the brain peripheries (approx. 10 µm; [10]).

Several MRI studies have confirmed the impact of pathology on brain tissue motion; pathologies explored to date include brain tumours [11], idiopathic intracranial hypertension [5] and Chiari malformation 1 [12]. In one patient with a meningioma, mean maximal caudal velocity was found to be abnormally low (≤0.3 mm s^−1^) when compared with values identified in healthy volunteers (0.5–1.5 mm s^−1^; [11]). Idiopathic intracranial hypertension patients exhibited significantly lower mean pontine displacement (0.06 mm [s.d.: 0.04]) compared with controls (0.11 mm [s.d.: 0.03]), *p* = 0.01. This difference decreased following an intervention to relieve the pressure, with mean pontine displacement increasing by 0.02 mm (s.d.: 0.03) post-lumbar puncture. A Doppler-based technique, known as tissue pulsatility imaging (TPI), has also been used to measure BTP [13–20]. TPI studies suggest that BTP changes may be associated with psychiatric conditions, such as depression [16,19–21]. A recent study by Desmidt *et al.* [20] identified TPI as a potential marker for treatment response in depressive patients exposed to an equimolar mixture of oxygen and nitrous oxide.

## 3. Brain tissue velocimetry

This paper documents the development of a clinical TCTD ultrasound prototype for estimating brain tissue motion and monitoring cerebral haemodynamics, named Brain TV. This was developed by Nihon Kohden, Japan, in collaboration with researchers from the University Hospitals of Leicester NHS Trust and University of Leicester between 2014 and 2024. Brain TV gathers and analyses TCD ultrasound data to extract information about tissue velocities at multiple depths along a single beamline. Velocity estimates are converted to estimates of tissue displacement through integration and are down-sampled to 500 Hz to facilitate continuous monitoring of tissue motion in parallel with external physiological measurements. For raw TCD recordings obtained from multiple channels at a sampling frequency of 14 kHz, data storage demands would be prohibitive in a routine setting.

To inform the development of Brain TV, it was first necessary to confirm that estimates of brain tissue displacement could be extracted from traditionally acquired TCD recordings. Manufacturers typically ‘filter out’ low-velocity contributions to the Doppler spectrum due to tissue and probe motion. Tissue scatters ultrasound more strongly than blood, so filtering out this high-intensity low-velocity component from tissue improves the appearance of the Doppler spectrogram originating from red blood cells. Indeed, as the ultrasound probe is usually hand-held, motion artefacts would be a significant issue without filtering. By filtering out the tissue signal, TCD manufacturers are discarding potentially informative data regarding tissue motion. There is currently insufficient data to establish whether TCTD monitoring of tissue motion might prove clinically useful or add additional information compared with existing physiological monitoring techniques.

Following discussions with Professor Kirk Beach, TCD recordings were obtained from healthy subjects using a commercial Spencer Technologies (Seattle, WA, USA) ultrasound system equipped with a standard Spencer Technologies 2 MHz ultrasound probe. The Spencer device was adapted to enable 8 s of raw (unfiltered) TCD data to be recorded and exported for further analysis. This pilot feasibility study immediately confirmed the importance of an appropriate probe fixation. Being a Doppler technique, TCTD is extremely sensitive to motion; even imperceptible movements of the probe in a hand-held examination will completely obscure the BTP signal. Although fixation systems already existed for TCD monitoring, these were found to be unsuitable for TCTD, and therefore a lighter, more wearable fixation system needed to be developed.

An interesting consequence of the signal from tissue being stronger than that of blood is that signals can be obtained from positions on the head unsuitable for TCD monitoring of blood flow. TCD is strictly limited to specific acoustic windows in the skull (temporal, ophthalmic or occipital) and requires a trained operator to identify a good window and then direct the ultrasound beam onto the targeted vessel. An advantage of TCTD is that signals can be obtained from any position on the head that permits a secure fixation. A further advantage is that obtaining a signal is much easier than for blood flow measurements, as there is no need to direct, or angle, the beam so that it intersects a vessel. The probe can be placed flat on the surface of the skin, and provided there is good acoustic coupling using ultrasound gel, a signal is easily obtained. This opens the potential for TCTD to be used by inexperienced operators, in contrast to extensive training and experience required to correctly identify blood flow signals in TCD monitoring of blood flow.

To enable the timing of pulsations to be compared to the cardiac cycle R−R interval, participants also underwent a 3-lead electrocardiogram (ECG) using a Life Scope monitor (Nihon Kohden, Japan). Limitations of adapting the Spencer system to provide tissue motion recordings were that measurements could not be visualized in real-time and recording duration was limited to a total of 8 s of data. The Brain TV measurement system was developed with the intention of allowing real-time visualization and recording of continuous data and direct comparison of TCTD data with external physiological measurements as inputs. Two ultrasound signals and multiple external analogue inputs can be simultaneously recorded using the Brain TV data acquisition prototype.

**Figure 1.**
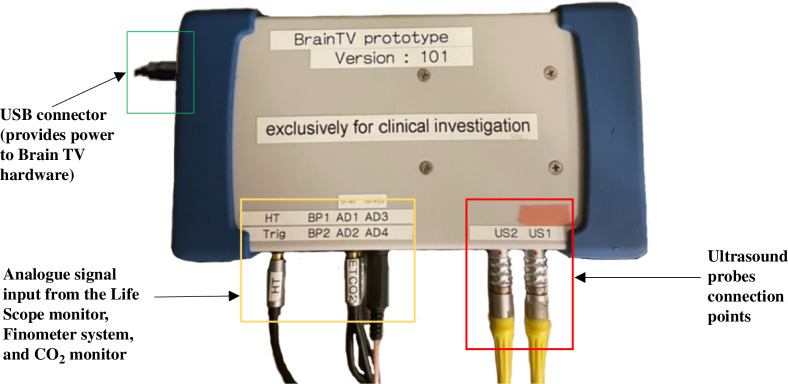
The Brain TV control unit. This captures brain tissue pulsation (BTP) recordings from two transducers and other physiological measurement data, including blood pressure (BP) and heart rate (HR), which are provided as inputs from an external device (e.g. Nihon Kohden Life Scope monitor, an OLG−3800 carbon dioxide monitor [Nihon Kohden, Japan], and a Finometer [Finapres Medical Systems B.V., Enschede, The Netherland]).

The Brain TV system has three components: a small control unit (figure 1), a pair of TCD probes (2 MHz piezoelectric single-element transducers) and a laptop PC or tablet for control and display. The control unit emits pulses of ultrasound with a central frequency of 2 MHz (as for standard TCD monitoring of blood flow). Brain TV was initially designed for bilateral, simultaneous monitoring, allowing synchronous BTP measurements to be obtained from two positions on the cranium. Initial tests used the same TCD transducers as supplied with the Spencer system. These are lightweight and easy to couple to the head using an elasticated headband and a custom-made probe holder. The system underwent extensive ultrasound output, electromagnetic compatibility and electrical safety checks prior to use in patients.

As with traditional TCD systems, the piezoelectric crystal converts ultrasound echoes into an electrical signal and an analog-to-digital converter is used to convert the electrical signal into a digital signal. Analysis of the backscattered echoes from tissue to identify the Doppler shifted (phase-shifted) components of the signal are then used to estimate tissue velocity in the direction of the ultrasound beam. Note that Brain TV velocity estimates will therefore be affected by Doppler angle in the same way as blood flow measurements. For a probe placed flat to the skin, TCTD estimates the component of tissue motion perpendicular to the skin’s surface.

Velocity estimates are then integrated to provide an instantaneous estimate of displacement. However, there are a couple of points to note when viewing the displacement waveforms. The first is that the displacement estimates are velocity-based; the sample volume is fixed, and the displacement estimate is based on velocity changes associated with tissue moving through this fixed volume. This differs from traditional methods of measuring displacement through speckle tracking, which offers far lower spatial and temporal resolution. The second point to note is that there is no set zero reference position.

In Brain TV, BTP measurements are obtained from 33 sample depths within the brain, ranging from 22 to 86 mm below the probe’s surface. Due to limited beam penetration, the deepest volumes may need to be discarded due to increased noise. Each depth provides a measurement of tissue velocity in the direction of the ultrasound beam from a cylindrical approximately 3 mm high × 5 mm diameter fixed sample volume. Adjacent depths are separated by 2 mm, resulting in a series of overlapping sample volumes.

## 4. Brain TV software

**Figure 2.**
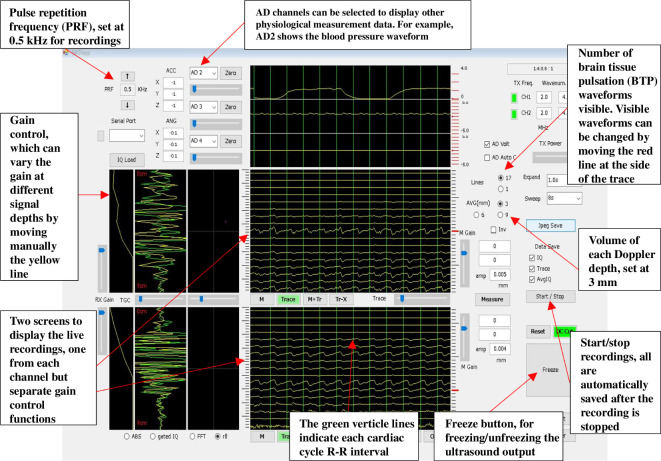
The Brain tissue velocimetry (Brain TV) user interface with labelled controls.

The Brain TV system connects to a Windows PC tablet (HP Elite X2 1012) using a universal serial bus connector cable on which the user interface software (NKDopp, developed in MATLAB (The MathWorks Inc., USA), Nihon Kohden) is installed. The user interface displays the BTP displacement signals in real time. It includes several control buttons that allow the recording channels to be turned on and off, adjustment of pulse repetition frequency, a freeze button to pause and resume data acquisition and a record feature to start and stop the recording (figure 2). Recordings are saved and analysed offline within a separate graphical user interface (GUI) developed in-house by Dr Andrea Lecchini-Visintini using MATLAB (R2022a; The MathWorks Inc., USA).

**Figure 3.**
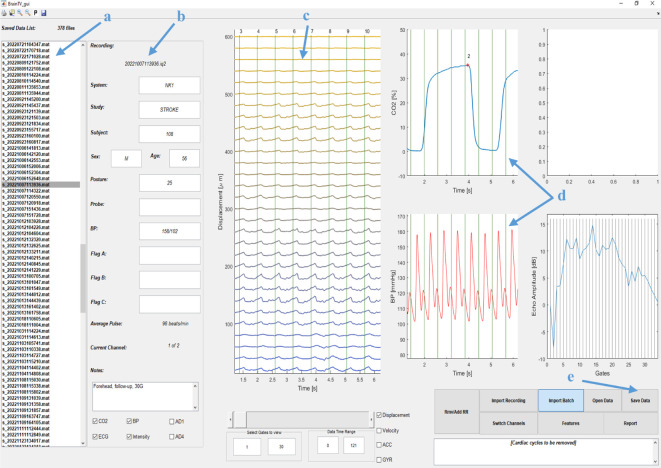
The Brain tissue velocimetry (Brain TV) data analysis graphical user interface (GUI). (*a*) saved Brain TV recordings, (*b*) recording file in use, (*c*) brain tissue pulsation (BTP) signals displayed to the user as a waterfall plot, (*d*) waveform data for end-tidal carbon dioxide (EtCO_2_) and (*e*) the save data button.

Data are then transferred to a data analysis GUI displayed in figure 3. The operator selects a data file of choice and the GUI then displays displacement of brain tissue over time at various depths as a waterfall plot (each line *y*-offset by 20 µm). The R–R interval is indicated by vertical lines and additional externally measured waveforms are displayed in adjacent windows. The operator is asked to manually input systolic and diastolic BP values from an arm-cuff measurement to calibrate the Finometer continuous BP data and for a BP waveform to be displayed. Waterfall plots and waveforms can then be visually inspected, with the R–R intervals overlaid. The operator reviews the number of depths where a clear signal has been obtained and can manually exclude intervals with motion artefacts from further analysis.

The GUI then computes features of the BTP displacement. Signal processing features, in which the most commonly used feature is peak-to-peak amplitude, are computed for BTP signals at each depth and at each cardiac cycle. Another BTP index used in the analysis is time-to-primary-peak, which is described as the time taken from the start of the cardiac cycle (beginning of the R–R interval) for peak displacement of BTPs to occur and is independent of the 'peak' being the lowest (nadir) or highest point. Features are also computed for a bulk BTP displacement signal, representing collective displacement, constructed as the average of BTP signals across all of the depths selected for analysis. Features are returned as a beat-to-beat time series in which each value is associated with the midpoint of the corresponding R–R intervals. Time averages of BTP features over selected intervals can then be computed and carried forward for analysis.

## 5. Ultrasound data processing

The Brain TV controller outputs in-phase and quadrature-phase (IQ) data from each TCTD recording, which are downsampled to 0.5 kHz to reduce file sizes, giving a temporal resolution of 2 ms. From these data, tissue velocity at each depth is estimated using the S-Dopp velocity estimator [22], which combines Doppler signals from adjacent subsample volumes, before computing a velocity estimate using an autocorrelation approach [23]. The S-Dopp velocity estimator reduces the variance of the velocity estimate with respect to the plain autocorrelation estimate. Tissue velocity estimates are then integrated over time to produce a BTP signal representing real-time displacement at each depth. BTP signals can then be optionally filtered using a bandpass filter to remove respiration and high-frequency noise.

## 6. Performance validation

The measurement performance of the Brain TV system was evaluated by Nihon Kohden using a soft biological tissue-mimicking material (TMM) phantom model. The ultrasound probe was attached to a high-resolution XY stage [YA05A, Kohzu Precision Co., Ltd.], with a resolution of 0.25 µm and repeat positioning precision less than or equal to ±0.3 μm. Water was used to couple the probe to the TMM phantom. The probe was repeatedly moved by 10–70 μm to measure the accuracy of Brain TV displacement measurements. This performance validation identified measurement errors of Brain TV to be less than 1.6 µm.

## 7. Brain TV set-up

**Figure 4.**
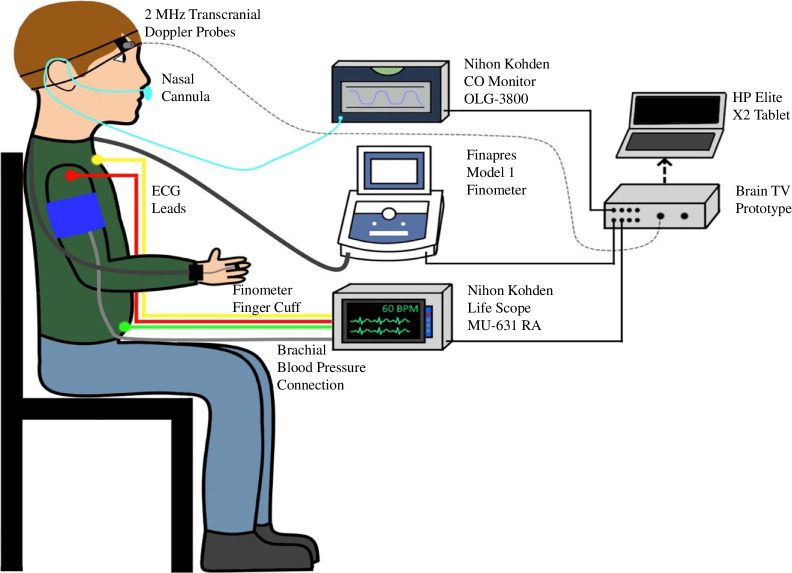
A schematic diagram to show the typical Brain tissue velocimetry (Brain TV) experimental setup. Participants may be seated (as shown) or lying supine or semi-supine (not shown).

Brain TV recordings were obtained using a pair of 2 MHz Spencer Technologies (Seattle, WA, USA) TCD probes. Once the Brain TV system is connected to an external 3-lead ECG and any other physiological measurement devices, simultaneous continuous recordings can be obtained. In our setup, a 3-lead ECG (Life Scope monitor, Nihon Kohden, Japan) was used to record the timing of the ECG wave R–R intervals. BP readings were obtained using a finger-cuff Finometer system (Finapres Medical Systems B.V., Enschede, The Netherland) attached to the participant’s left wrist, with an appropriately sized cuff positioned around the left middle finger. The Finometer was calibrated prior to each recording using a standard inflatable arm-cuff device. Capnography measurements of end-tidal carbon dioxide (EtCO_2_) were obtained using an OLG-3800 CO_2_ monitor (Nihon Kohden, Japan), connected to a nasal cannula. During recordings, participants were asked to remain still with their eyes closed to prevent motion artefacts. A typical Brain TV setup is demonstrated in figure 4.

## 8. BTPs in the healthy population

In order to characterize variations in brain tissue displacement in a healthy population, normative data were first gathered using an adapted Spencer Technologies TCD device (Seattle, WA, USA). Full details of this cross-sectional study are published in Turner [4]. The study gathered data from 107 healthy volunteers (56 men; mean age: 41 years, range: 20–81 years) and confirmed the feasibility of measuring BTPs using TCTD ultrasound in human subjects. This study also helped to establish normal values and explored factors such as age, sex, HR, mean arterial pressure (MAP) and pulse pressure (PP) that were likely to influence BTPs.

Out of 428 TCTD recordings, 405 were suitable for further analysis; 23 recordings were removed due to large numbers of artefacts. This study found that BTP amplitude varies considerably between individuals (~4 to ~150 µm). Regional and hemispheric differences in bulk BTP amplitude were identified, with median bulk BTP amplitude found to be significantly higher at the forehead position (17.0 µm [Inter-Quartile Range (IQR): 11.3–25.4]) than the temporal window (9.2 µm [IQR: 6.0–12.9]), *p* < 0.001. BTP amplitude was also found to increase approximately linearly with depth for both positions; the strongest BTPs were recorded deeper into the brain, which is consistent with MRI findings [10]. Stronger right hemisphere pulsations were measured in 59% of participants and 57% of participants at the forehead and temporal windows, respectively.

Multivariate regression modelling identified PP as a significant predictor of bulk BTP amplitude at both the forehead and temporal positions, with a 1% increase in PP resulting in a 0.8% and 0.6% increase in bulk BTP amplitude at the forehead and temporal positions, respectively. Age was also identified as a significant predictor of bulk BTP amplitude at the forehead and sex as a significant predictor at the temporal position. For every 1 year of age from the age of 20, bulk BTP amplitude decreased at the forehead by 0.9%, *p* < 0.001. Meanwhile, BTPs were 29% higher in men than women at the temporal window, *p* = 0.01.

This healthy volunteer study confirmed that BTPs can successfully be collected from multiple positions on the head and suggested patient factors that may influence BTP amplitude. These healthy population data may provide a useful reference for comparison with patient data in future research.

It has previously been suggested that mild hypocapnia may occur in patients with AIS [24], and that hypocapnia increases BTPs [25]. To confirm the effect of hypocapnia on BTPs, a hyperventilation task was used to reduce EtCO_2_ by 5 mmHg in 30 healthy participants (12 women), with a median age of 25 years (IQR: 24–30). This was the first physiological measurement study to use the Brain TV system for continuous measurement of BTPs. The findings of this study are published in Alharbi *et al.* [26]. During analysis, it was found that the Brain TV device had a more limited (6 cm) penetration depth for this study compared with 8 cm for the Spencer device, reducing the number of depths suitable for analysis to 20.

Hyperventilation successfully induced a 5.0 mmHg drop in EtCO_2_ [95% CI −6.2, −3.9; *p* < 0.0001] and was accompanied by slight increases in the median bulk BTP amplitude of 1 µm in the left hemisphere and 1.6 µm in the right hemisphere. These small increases in BTP were much smaller than the baseline variability in BTPs between subjects (approx. 4 to 45 µm). Potential confounders included MAP, which decreased in the cohort by 3.5 mmHg [95% CI −5.2, −1.8; *p* = 0.0002], whilst HR increased by 4.4 bpm [95% CI 1.7, 7.1; *p* = 0.002]. These changes were found to be statistically significant during hyperventilation at an adjusted *p*-value of *p =* 0.004. PP increased in the cohort by 4.8 mmHg, but this change was not statistically significant.

During recovery, EtCO_2_ significantly increased by 3.5 mmHg in the cohort [95% CI 2.5, 4.5; *p <* 0.0001]. This increase corresponded to a significant decrease in bulk BTP amplitude in both brain hemispheres; in the right hemisphere, the median bulk BTP amplitude dropped by 4.3 µm, *p =* 0.001, and in the left hemisphere, the median bulk BTP amplitude dropped by 1.3 µm, *p =* 0.02. Potential confounding factors included MAP, which significantly increased in the cohort in recovery by 3.3 mmHg, *p* = 0.002, whilst HR significantly decreased below baseline in recovery by 6.0 bpm [95% CI −8.6, −3.4; *p* < 0.0001]. Although bulk BTP amplitude decreased during recovery, changes were small, ranging from ~1 to ~4 µm in the cohort. This is far smaller than the variability in bulk BTP amplitude observed between subjects.

Based on the results of this experiment, challenges involved in systematically exploring cerebrovascular physiology in the presence of confounders and high baseline variability became apparent. Overall, this study identified that mild hypocapnic changes have little impact on BTP measurements.

## 9. BTPs in pathology

To explore whether BTPs are disturbed in the presence of AIS, a proof-of-concept study using the Spencer Technologies system was performed in 14 AIS patients (11 female, of median age 74 years [IQR: 63–81]) with mild to moderate stroke severity. Findings were compared to 24 age-matched non-stroke controls (13 female, of median age 68 years [IQR: 58–74])[4]. Detailed information on the study is provided by Ince *et al.* [27].

Estimates of tissue displacement were recorded from 30 depths through the left and right forehead in stroke patients and non-stroke controls. This resulted in 38 pairs of 8 s TCTD recordings for review. No significant differences were identified between affected and unaffected hemispheres in the AIS cohort. However, the AIS group had significantly weaker combined bulk BTP amplitude compared to non-stroke controls. Combined (right and left hemisphere) median bulk BTP amplitude in the AIS cohort was 12 μm [IQR: 10−14]) compared to a value of 17 μm [IQR: 11−24]) for the non-stroke controls, *p* = 0.03. Potential confounders in the AIS group included a significantly greater median PP of 77 mmHg [IQR: 67–99] compared to 48 mmHg [IQR: 43–60] in the non-stroke controls, *p* < 0.001. No statistically significant differences were identified in HR or MAP.

**Figure 5.**
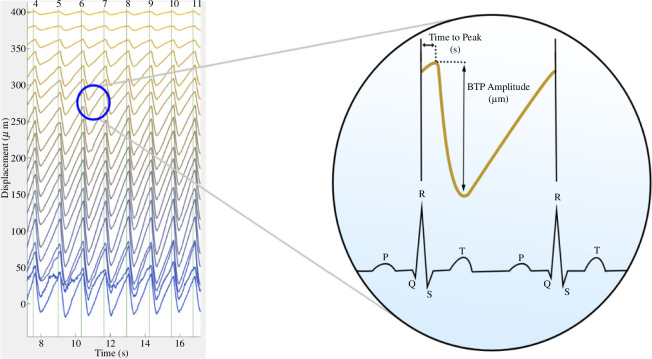
A Brain tissue velocimetry (Brain TV) recording is displayed in MATLAB; a waterfall plot is produced where the signal from adjacent depths is offset by 20 µm. Horizontal lines correspond to the depths in the brain from below the probe’s surface. Depth 1 is displayed as ~22 mm below the probe’s surface at the top (yellow) and depth 20 displayed as ~60 mm (blue). Vertical lines indicate the R-R interval for each cardiac cycle and are numbered at the top of the waterfall plot. A depiction of time-to-primary-peak (TtPP) and brain tissue pulsation (BTP) amplitude are given across the cardiac cycle.

Qualitative analysis of forehead BTP waterfall plots by three expert observers confirmed that the healthy volunteer BTP waveforms exhibited a typical waveform shape that peaks soon after the start of the R-R interval, followed by a short period of motion away from the probe and then a sharp upturn in systole. An example of this is given in figure 5. In the stroke subjects, disruption to the waveform shape was identified, such as additional peaks and oscillations, an absence of clear pulsations (or increased noise) and poorly correlated signals (characterized by spatial and temporal variability), particularly in the affected hemispheres.

This study was the first to record brain tissue motion measurements from stroke patients to provide an initial qualitative exploration of BTP waveform features. TCTD data from 14 AIS patients was successfully recorded, suggesting measurements are feasible and well tolerated. However, quantitative results should be interpreted with caution; high baseline variability in BTPs between subjects and small sample sizes mean that underpowered studies can yield erroneous results. Despite a significantly higher than average PP (1.6 times) in the AIS cohort, BTPs were lower in magnitude, which warrants further investigation. Further stroke studies would also provide the opportunity to include other stroke sub-types, such as haemorrhagic stroke.

## 10. BTPs in phantom models

Although PP was identified as a significant predictor of BTPs in a healthy population, the effect of MAP on BTPs required further clarification. An ultrasound brain phantom model was developed to generate artery-induced BTPs under controlled conditions, as described by Nicholls *et al.* [28]. This phantom was also used to help understand the impact of major cerebral artery pulsations on brain motion using Brain TV. Two separate experiments were performed to study the impact of PP and MAP on bulk BTP amplitude. BTP signals were successfully obtained from 27 depths during the MAP experiment and 30 depths during the PP experiment within the phantom.

Increasing MAP from 86 to 228 mmHg resulted in a slight increase in PP of 6 mmHg and corresponded with a slight increase in bulk BTP amplitude of ∆BTP = +0.3 µm, from 18.3 to 18.6 µm. Increasing PP from 12 to 101 mmHg was accompanied by an increase in MAP of 41 mmHg. These changes in BP resulted in a significant increase in bulk BTP amplitude (measured from 27 depths) of 24.5 µm, from 3.7 to 28.2 µm. A regression model confirmed that each 1 mmHg increase in PP generated an increase in bulk BTP amplitude of 0.29 µm (*R*^2^ = 0.978).

The results of this study suggest that cerebral arterial PP has a significant impact on BTP amplitude, whereas MAP has no discernible impact. However, the TMM brain was not surrounded by CSF under pressure and therefore may not replicate the more complex coupling between arterial PP, ICP and tissue motion when constrained within the skull. The walls of the arterial replica were also thicker (approx. 1 mm) and less elastic than real cerebral arteries, which may have reduced transfer of arterial pulsations into the tissue. The development of more realistic phantoms simulating raised ICP or large vessel occlusion (LVO) may help in understanding factors affecting BTPs through further experiments.

## 11. Future perspectives

For the design of future studies using TCTD, it would be useful to standardize acquisition and analysis methods. For example, there are several ways of computing aggregate measures, and as BTP amplitude is known to vary regionally and with depth, differences in probe position and penetration depth are likely to influence motion estimates. It may also be worth reaching a consensus on whether to discard shallower depths, such as 10 to 20 mm, since their displacements are typically low. A machine learning framework and data-sharing arrangements may help to inform the selection of aggregate measures. Looking beyond waveform amplitude, additional signal-processing waveform features could also be explored. A library of waveform features could be defined in a similar way as in established ECG and electroencephalogram analysis procedures [29,30]. The optimal approach to analysis might also differ with probe location, corresponding to the different regions being probed. The data outlined in this article hints that waveform changes are more likely to signal pathology than pulsation amplitude metrics, which vary widely within and between individuals.

It would be desirable to create a reference map that links surface probe positions to underlying brain anatomy, allowing for accurate registration of sensor locations to a head atlas. It may be possible to adapt recent approaches developed for functional near-infrared spectroscopy (fNIRS; [31,32]), incorporating information on the shape of each subject’s head to improve accuracy of the registration procedure.

The use of Brain TV as a diagnostic tool is still largely unexplored. One promising application is non-invasive ICP monitoring in intensive care units, where Brain TV’s small and portable design could be used for continuous bedside monitoring, potentially reducing unnecessary neurosurgical interventions and enabling prompt treatment. Another promising area could be pre-hospital diagnosis of LVO stroke. Due to its portability, the device could be easily used by clinicians in an ambulance to identify LVOs early, facilitating direct transfer to specialized centres for mechanical thrombectomy, which could significantly improve patient outcomes [33]. Brain TV may also supplement confirmatory neuroimaging while patients await their scan results, providing information on waveform variability, including the pulsation shape, pulsation amplitude and the frequency of pulsations, but further work is required to confirm this.

The studies reviewed in this article focused on pulsations generated by the heart. Other sources of movement were removed by a band-pass filter that eliminates both high-frequency noise and low-frequency content. Vasomotion, found at lower frequencies of around 0.1 Hz in humans, is also of interest, as this is linked to the glymphatic system [34,35]. Exploring the low-frequency range, especially in connection to sleep, is an open task.

Moreover, it would be useful to validate TCTD displacement data with complimentary MRI-based techniques, such as amplified magnetic resonance imaging and displacement encoding with stimulated echoes [36]. This will require performing measurements on the same subjects in the same experimental session using both modalities for comparison and validation.

Finally, building and sharing a database of recordings is an important next step. Brain TV’s capacity for large-scale data collection across numerous subjects and repeated measurements makes it an efficient tool for building an extensive dataset. If properly annotated, such a dataset can support the development and validation of high-fidelity digital twins of brain mechanics in both health and disease. It would provide a valuable resource for informing models of the brain, enabling more accurate modelling, simulation and ultimately, better clinical outcomes.

## Data Availability

This article has no additional data.
